# Genetic Variation and Immunohistochemical Localization of the Glucocorticoid Receptor in Breast Cancer Cases from the Breast Cancer Care in Chicago Cohort

**DOI:** 10.3390/cancers13102261

**Published:** 2021-05-13

**Authors:** Umaima Al-Alem, Abeer M. Mahmoud, Ken Batai, Ebony Shah-Williams, Peter H. Gann, Rick Kittles, Garth H. Rauscher

**Affiliations:** 1 Division of Epidemiology and Biostatistics, University of Illinois at Chicago, Chicago, IL 60612, USA; ualale2@uic.edu (U.A.-A.); garthr@uic.edu (G.H.R.); 2 Department of Medicine, College of Medicine, University of Illinois at Chicago, Chicago, IL 60612, USA; 3 Department of Urology, The University of Arizona, Tucson, AZ 85724, USA; kbatai@email.arizona.edu; 4 Medical & Molecular Genetics, School of Medicine, Indiana University, Bloomington, IN 46202, USA; eshahwil@iu.edu; 5 Department of Pathology, University of Illinois at Chicago, Chicago, IL 60612, USA; pgann@uic.edu; 6 City of Hope Comprehensive Cancer Center Duarte, Duarte, CA 91010, USA; rkittles@coh.org

**Keywords:** breast cancer, glucocorticoid receptor, cytokeratin 5/6, psychological stress, genetic ancestry, single nucleotide polymorphism, immunohistochemical localization, multispectral digital imaging, hormonal receptor, molecular subtypes, basal-like breast cancer, estrogen receptor, progesterone receptor, tissue microarrays

## Abstract

**Simple Summary:**

Breast cancer, one of the leading causes of death among women, is a complex disease in which several factors, such as psychosocial stress, have been implicated in its initiation and progression. The glucocorticoid receptor (GCR) is one of the molecules that transfers the stress signal into the body. We measured the genetic variation and protein expression of GCR and the genes that regulate GCR function or response and examined whether these variations were associated with breast cancer. We found several genetic variants of functionally important SNPs associated with later disease stage, higher grade, and hormone receptor-negative status. The GCR protein expression was reduced in breast cancer tissue and correlated with the basal cell marker CK5/6.

**Abstract:**

Background: Glucocorticoid, one of the primary mediators of stress, acts via its receptor, the glucocorticoid receptor (*GCR/NR3C1*), to regulate a myriad of physiological processes. We measured the genetic variation and protein expression of GCR, and the genes that regulate GCR function or response and examined whether these alterations were associated with breast cancer clinicopathological characteristics. Method: We used samples from a multiracial cohort of breast cancer patients to assess the association between breast cancer characteristics and the genetic variants of single nucleotide polymorphisms (SNPs) in *GCR/NR3C1*, *FKBP5*, *Sgk1*, *IL-6*, *ADIPOQ*, *LEPR*, *SOD2*, *CAT*, and *BCL2*. Results: Several SNPs were associated with breast cancer characteristics, but statistical significance was lost after adjustment for multiple comparisons. GCR was detected in all normal breast tissues and was predominantly located in the nuclei of the myoepithelial cell layer, whereas the luminal layer was negative for GCR. GCR expression was significantly decreased in all breast cancer tissue types, compared to nontumor tissue, but was not associated with breast cancer characteristics. We found that high nuclear GCR expression was associated with basal cell marker cytokeratin 5/6 positivity. Conclusion: GCR expression is reduced in breast cancer tissue and correlates with the basal cell marker CK5/6.

## 1. Introduction

Breast cancer, one of the leading causes of death among women, is a complex disease in which genetic, epigenetic, and environmental factors have been implicated in its initiation and progression. Psychosocial stress may play a role in the etiology of breast cancer, but the literature is conflicting. Few studies have found a positive association between psychosocial stress and the risk of having breast cancer [1,2]; other prospective and retrospective studies have yielded conflicting findings, with the majority of studies reporting no association [3,4,5,6,7], and even the reverse relationship [6,8]. Furthermore, systematic reviews and meta-analyses of these studies [9,10,11,12] were equivocal. A limitation in the literature is the lack of epidemiological studies attempting to link psychosocial factors to biologically plausible intermediates. Although further downstream signals converting psychosocial stress into cellular dysregulation and finally into breast cancer are not well understood, animal and in vitro studies have implicated glucocorticoid hormones in this process [13,14,15,16,17,18].

Glucocorticoids play an important role in several cellular processes, including apoptosis, inflammation, mammary development, and tumorigenesis [19]. Glucocorticoid signaling is mediated through the functional isoform, glucocorticoid receptor-alpha that resides predominantly in the cytoplasm. GCR is expressed in almost all human tissues in a cell-specific manner [20,21]. GCR activity is modulated by its level, subcellular localization, and interactions with other genes. Altered GCR response has been associated with the pathogenesis of several diseases, such as altered susceptibility to sporadic breast-cancer among Caucasian women [22], metabolic syndrome [23], cardiovascular disease [24], rheumatoid arthritis [25], and depression [26].

GCR is predominantly expressed in myoepithelial cells [27,28,29,30] in normal breast tissue and all stages of breast cancer; however, the relationship between breast cancer progression and GCR expression and subcellular localization appears inconsistent. A wide range of GCR levels (0 to 90% positive cells) in the cytoplasmic and or nuclear compartments has been reported previously in breast cancer tissue [27,28,29,30].

The purpose of this study was to examine the association between breast cancer characteristics and GCR in a series of breast cancer cases with defined clinical and histological characteristics, as we hypothesize that these alterations would be associated with breast cancer subtype or aggressiveness. We examined the association between breast cancer characteristics and genetic variants in *GCR/NR3C1* and genes downstream of GCR activation: *FKBP5*, *Sgk1*, *IL-6*, *ADIPOQ*, *LEPR*, *SOD2*, *CAT*, and *BCL2*. To investigate GCR protein expression and subcellular localization, we used tissue microarray arrays (TMA) and multispectral digital imaging.

## 2. Materials and Methods

### 2.1. Study Population and Biological Samples

Patients and samples for this study are from the Breast Cancer Care in Chicago (BCCC) study, conducted by the UIC Center for Population Health and Health Disparities (NCI P50 CA106743). BCCC is a population-based cross-sectional study of women diagnosed with primary invasive breast cancer cases between 1 October 2005, and 29 February 2008 [31]. The parent study protocol was approved by the University of Illinois at Chicago Institutional Review Board (IRB#2010-0519). DNA samples and paraffin-embedded surgical samples were obtained from diagnosing hospitals prior to radiation or pharmacotherapy. Description of the BCCC cohort had been previously reported [32]. We had 656 cases with valid genetic ancestry estimates and linked clinical, sociodemographic, and epidemiological data to assess candidate gene variance. Tumor tissue from the invasive component from 287 cases was available for the immunohistochemical study (IHC).

### 2.2. SNP Selection and Genotyping

We genotyped 59 functionally important SNPs and tagging SNPs in GCR and GCR-associated genes. The SNPs were selected based on a minor allele frequency greater than 5% and previous association with GCR activity, breast cancer, or downstream related pathways such as inflammation and apoptosis. Genotyping was performed with iPLEX Gold assay on a MALDI-TOF (matrix-assisted laser desorption/ionization time-of-flight) mass-spectrometer (MassArray system) according to the manufacturer's recommendations. Genotyping quality control for all SNPs was assessed using blinded duplicate genotyping for 60 DNA samples. A genotype concordance rate of 99% was observed for all markers. Genotyping call rates exceeded 98.5% for all individuals included in the analyses.

### 2.3. Self-Reported Race/Ethnicity and Genetic Ancestry with Ancestry Informative Markers (AIMS)

Race and ethnicity were each defined at the interview through separate self-identification of Hispanic ethnicity and race. Racial/ethnic groups were categorized as non-Hispanic White, non-Hispanic Black, and Hispanic. Global genetic ancestry for the BCCC cohort was previously reported [32]. Ten cases that self-reported as non-Hispanic White and had more than 70% West African genetic ancestry were excluded. After the exclusions, genotype information was available for a total of 656 cases.

### 2.4. Tissue Microarray Immunohistochemical Staining and Scoring

Three tissue microarrays (TMA) were constructed from the BCCC breast cancer cases subcohort and stained as previously described [33]. The TMA consisted of tumor tissue for 287 cases and 26 normal breast tissues from unaffected women obtained by reduction mastectomy procedures and five fibroadenomas. A list of antibodies for immunohistochemical staining is summarized in Table A1. Immunohistochemical staining for GCR, performed by the UIC Research Histology and Tissue Imaging Core facility, was optimized by testing different sources and dilutions of the primary antibody and different antigen retrieval methods. Manual and digital scoring was performed as previously described [33]. GCR expression was evaluated based on the percentage of positive tumor cells and staining intensity. H-scores were calculated as the sum of staining intensity (0,1,2,3) and the percentage of cells (0–100%) in each intensity category (0, 1+, 2+ and 3+). The final scores were on a continuous scale between 0 and 300. An average H-score of the triplicate cores was used during analysis. 

### 2.5. Statistical Analysis

Baseline characteristics of the population were compared across self-reported racial/ethnic groups using χ2 test for categorical variables and ANOVA for continuous variables. The primary response variable was GCR expression. GCR expression was dichotomized at the median to assess association with our outcome variables: stage at diagnosis, hormone receptor status, and histologic grade as markers of breast cancer progression or aggressiveness. The stage at diagnosis was categorized using the American Joint Committee on Cancer (AJCC) categories (0–4), with the later stage at diagnosis defined as stage >2 versus stage <1. Histological grade was determined through the Nottingham grading system. The higher grade was defined as grade intermediate and high versus low. ER/PR status was defined as positive if the tumor contained ER and/or PR receptors and negative in the absence of both receptor types. Molecular subtypes were categorized as Luminal A, Luminal B, HER2+, and triple negative. We also fitted logistic regression models to estimate the odds ratios (OR) and 95% confidence intervals (CIs). All reported *p*-values are two-sided, and a p-value < 0.05 was considered statistically significant. Statistical analyses were conducted using Stata version 11 (College Station, TX, USA). 

For each SNP, the deviation of genotype frequencies from Hardy−Weinberg equilibrium (HWE) was assessed using χ2 test. The homozygous wild-type genotype served as the reference category. Association analyses were performed under dominant, recessive, or additive modes of inheritance. Separate logistic regression models were run for each self-reported racial/ethnic group (White, Black, and Hispanic), ancestry (European, West African, and Native American), and tumor characteristic to estimate OR (95% CI). We performed separate analyses for each racial/ethnic group because of the potential biological and environmental differences in factors contributing to breast cancer. The regression models were adjusted for health insurance, income, education, nulliparity, and age at first and last birth. All reported p-values are two-sided. A Bonferroni correction was used to account for multiple comparisons. Statistical analyses were conducted using R-Studio and Stata version 11 (College Station, TX, USA).

## 3. Results

### 3.1. Baseline Characteristics of the BCCC Sub-Cohort for the Genetic Study

The final cohort’s tumor and demographic characteristics included 250 White, 273 Black, and 120 Hispanic women (Table 1). The mean age at diagnosis was 55 years (range 25 to 78 years). Black and Hispanic women were diagnosed at a later stage, with higher grade disease and a higher proportion of ER/PR negative tumors than Whites. In addition, a greater proportion of Black and Hispanic women were overweight/obese, had more co-morbidities, were less likely to have their cancer detected through screening mammography, had a lower level of education and income, and less likely to have private insurance than Whites. The predominant genetic ancestry proportion among White cases was European genetic ancestry, with a mean of 90% (±SD 11%). The predominant genetic ancestry among Black cases was West African genetic ancestry, with a mean of 80% (±SD 13%). Hispanic women had a wide range of European (mean 40%), Native American (mean 40%), and West African (mean 20%) genetic ancestry representing a highly admixed group. 

### 3.2. Characteristics of Studied Markers

In the current analysis, we examined polymorphisms in *GCR*, *Sgk1*, *BCL2*, *FKBP5*, *IL6*, *ADIPOQ*, *LEPR*, *SOD2*, and *CAT*. The polymorphisms, including the minor allele frequencies (MAF) and HWE results by self-reported race/ethnicity summarized in Table A2. SNPS that failed the MAF and HWE (*p* = 0.05) in each self-reported racial/ethnic group were removed (Table A3). 

We observed different allelic frequency distributions between the racial/ethnic groups for several SNPs (*GCR*: rs6191, rs33388, rs9324924, rs4607376; *Sgk1*: rs9493857; *BCL 2*: rs2279115; *LEPR*: rs1137101; *SOD2*: rs4880). Our reported allele frequencies were similar to those in the Single Nucleotide Polymorphism Database [34]. A Bonferroni correction was used to account for multiple comparisons. There were 52 comparisons for the Blacks category with a corrected alpha = 0.001 and 49 comparisons for Whites and Hispanics with a corrected alpha: 0.001. None of the associations between those SNPs and breast cancer characteristics remained statistically significant after adjustment for multiple comparisons.

### 3.3. Genotypes and Histological Grade at Diagnosis

Table 2 summarizes the significant associations (*p* < 0.06) between higher histological grade at diagnosis and individual SNPs. Among the White cases, a higher grade at diagnosis was associated with the *GCR* rs33388_TT_ and rs6191_GG_ genotypes were associated with two-fold increased odds of high-grade disease. The *GCR* rs41423247_GC+CC_ genotype was associated with lower grade disease (OR 0.56: 95% CI 0.32–0.99). IL-6 rs1800797_AG+AA_ genotype (OR, 1.99:95% CI 1.07–3.73) was associated with higher grade. Among the Black cases, *GCR* rs10052957_AG+AA_, rs258813_AA_, rs2918418_AA_, rs33388_AA_, rs41423247_GC/CC_, rs6188_TT_, rs6191_TT_ and rs9324924_GG_ genotypes were associated with higher grade disease, whereas *GCR* rs10482616_GA+AA_, rs10482672_TC+TT_, or rs7701443_AG+GG_ or rs9296158_AA_ genotypes were associated with lower grade disease. The *FKBP5* rs9296158_AA_ genotype was associated with lower grade disease (OR 0.45: 95% CI 0.23–0.9). Among the Hispanic cases, only the *GCR* rs9324924_GT+TT_ genotype was associated with higher grade disease (OR 3.14: 95% CI 0.99–10). None of the associations between those SNPs and stage at diagnosis remained statistically significant after adjustment for multiple comparisons.

### 3.4. Genotypes and Stage at Diagnosis

We examined the association between later stage at diagnosis and individual SNPs among breast cancer cases (Table 3). Among Black cases, the A allele of *GCR* rs10482614 was associated with later stage at diagnosis (OR, 8: 95% CI 2–39), but there were few cases (*n* = 12) in this category. Several SNPs in the *FKBP5* gene were associated with stage at diagnosis. Black cases with *FKBP5* (rs3777747_GG_) were associated with a later stage of diagnosis (OR, 2:95% CI 0.98–4.11). However, the *FKBP5* genotypes rs3800373_GT+GG_, rs9296158_AG+AA_, 9470080_CT+TT_ were associated with a nearly 50% decreased prevalence of the later stage at diagnosis. For Hispanic cases, the ADIPOQ rs1501299_CA+AA_ genotype was associated with decreased odds of later stage (OR, 0.39: 95% CI 0.17–0.87), while the rs266729_GC+GG_ genotype was associated with late stage (OR, 3.01: 95% CI 1.35–6.73). None of the tested SNPs were statistically significant at *p* < 0.06 level for White cases. None of the associations between the studied SNPs and grade at diagnosis remained statistically significant after adjustment for multiple comparisons.

### 3.5. Genotypes and Hormone Receptor Status

Table 4 summarizes the results of the significant association (*p* < 0.06) between ER/PR positivity and individual SNPs. Among White cases, we found an inverse association for the CC genotype of *GCR* rs12656106 and ER/PR positivity (OR, 0.47; 95% CI 0.17–1.35). For Black cases, *GCR* (rs10482616*_GA+AA_*), *ADIPOQ* (rs1501299_CA+AA_) and *BCL2* (rs2279115_AA_) were associated with ER or PR receptor positivity. None of the SNPs were significant at alpha < 0.06 among Hispanic cases. Overall, none of the associations between those SNPs and hormone-receptor status remained statistically significant after adjustment for multiple comparisons. All the significant results (*p* < 0.06) are summarized in Table 5.

### 3.6. Characteristics of the BCCC Subcohort for the TMA Study

We measured GCR protein expression in breast cancer tissue from 287 cases. The descriptive statistics of this subset are summarized in Table A4. The mean age at diagnosis was 56 years (SD ± 11), and cases consisted of 103 Black, 84 White and 80 Hispanic patients. The cases in the subcohort still showed the racial/ethnic disparity in the distribution of patient characteristics. A greater proportion of Black and Hispanic women were overweight/obese, had more comorbidities, were less likely to have their cancer detected through screening mammography, had a lower level of education and income, and less likely to have private insurance than Whites. Black women were diagnosed at a later stage, with higher grade disease. Most of the cases were of the ductal histological type, luminal A molecular subtype, and were ER and/or PR positive (Table 6).

### 3.7. GCR Expression and Subcellular Localization in Normal and Cancer Tissue

**Figure 1:**
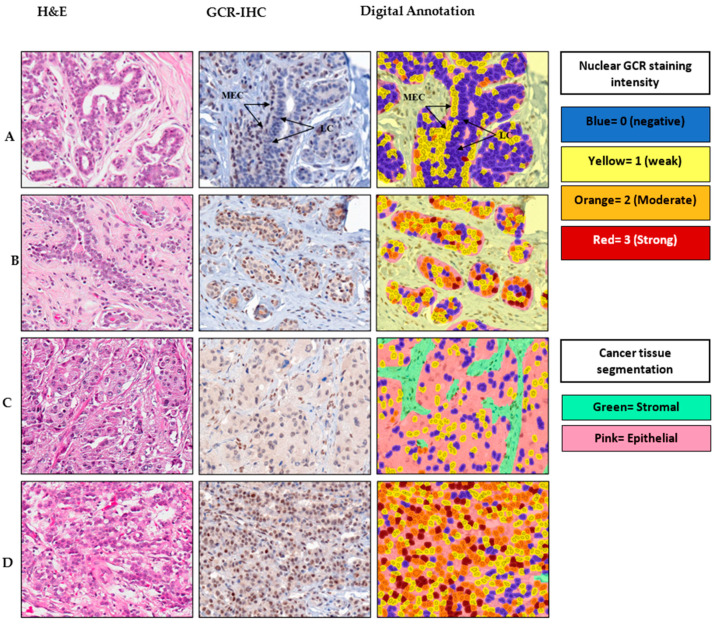
Immunohistochemical staining and digital output images for glucocorticoid receptor (GCR) in representative cases. For each case, H&E and the corresponding anti-GCR IHC image and digital analysis output are shown. (**A**) Normal breast tissue, (**B**) fibroadenoma, (**C**) ductal carcinoma and (**D**) lobular carcinoma. LC, luminal cells; MEC, myoepithelial cells.

Representative images of nuclear GCR staining intensity in normal, fibroadenoma, and cancerous breast tissue, along with digital imaging annotation, are shown in Figure 1. In normal breast tissue, GCR was expressed predominantly in the nuclei of the myoepithelial cell layer that surrounds normal ducts and lobules. The luminal layer in normal breast tissue was negative for GCR. Among the fibroadenoma samples, GCR staining was not limited to the myoepithelial layer as nuclear and cytoplasmic staining of luminal epithelial cells was also detected. There was diffuse GCR staining throughout the cancer foci in the breast cancer tissue, which was likely due to the loss of normal glandular architecture and outlining myoepithelium in these malignant cells. 

### 3.8. GCR Expression in Normal and Cancer Tissue Using Digital Scoring

GCR was detected in both the cytoplasm and nuclear compartments of the normal myoepithelial cells and the GCR-positive breast cancer cells. Despite the low expression of GCR in the cytoplasm relative to the nuclear compartment, there was a strong correlation between nuclear and cytoplasmic H-scores. (Spearman’s Rho = 0.80; *p* < 0.00001 and r^2^ = 0.72).

GCR staining was lower in cancer tissue compared with normal tissue and fibroadenoma samples. When we dichotomized nuclear H-scores for breast cancer cases at the sample median for all samples (mean H-score = 17), 44% of breast cancer cases had positive nuclear staining as opposed to 100% in normal breast tissue and fibroadenoma, respectively (Table 6). In breast cancer tissue, cytoplasmic staining (mean H-score = 3) was weaker than nuclear staining (mean H-score = 29); 57% of breast cancer TMA cores had an H-score = 0 for cytoplasmic GCR. We did not observe a statistically significant difference in GCR staining among breast cancer subtypes. However, compared with ductal carcinoma, lobular carcinoma had greater nuclear GCR expression (mean H-score: 27 s. 36, respectively) and a greater percentage of nuclear positive cases (42% versus 48%, respectively). 

### 3.9. Correlation between Nuclear GCR Expression and Breast Cancer Characteristics

We measured the association of GCR staining with clinicopathologic characteristics, histological and molecular breast tumor subtypes. Positive nuclear GCR expression was weakly associated with any strong family history of breast cancer (*p* = 0.069) but was not associated with self-reported race, BMI, nulliparity, menopausal status, stage or grade at diagnosis, or subtypes of breast cancer. 

### 3.10. Correlation between Nuclear GCR and CK 5/6 Expression

**Figure 2:**
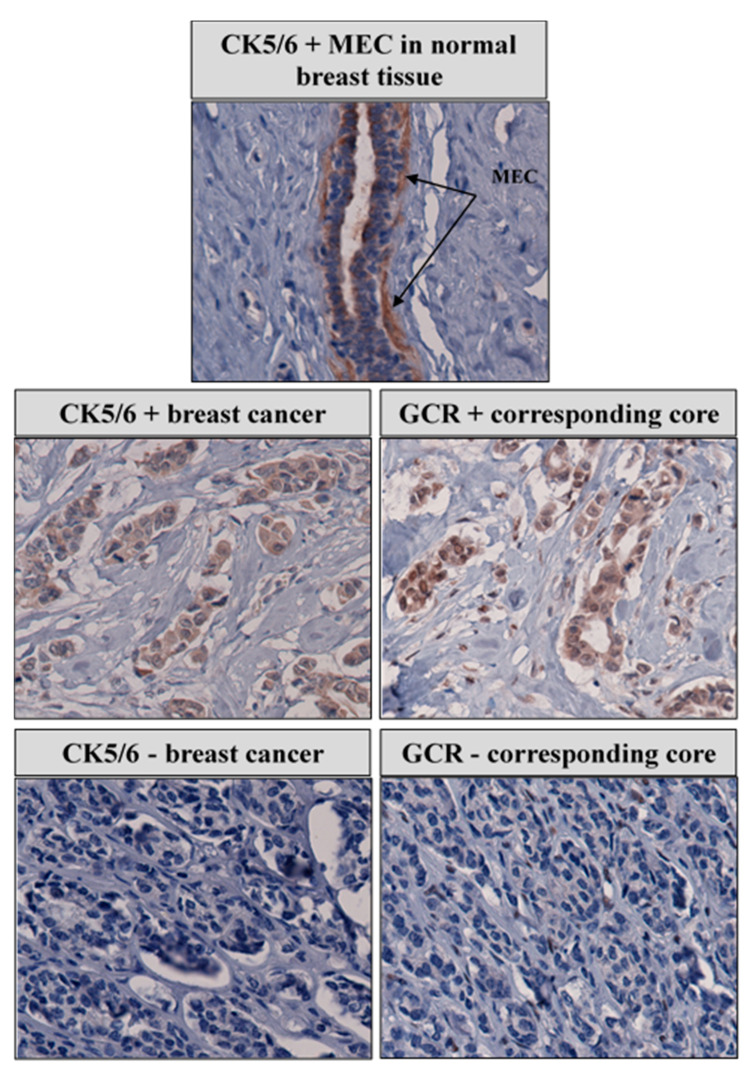
Correlation between GCR and CK 5/6 and expression in representative breast cancer cases.

In our immunohistochemical study, nuclear GCR staining strongly correlated with cytoplasmic CK 5/6 expression, a marker of the tumor's basal nature. Figure 2 is a representative staining pattern of CK 5/6 in nontumor and breast cancer tissue, illustrating the correlation between GCR and CK 5/6. We observed diffuse cytoplasmic staining of CK 5/6 in the myoepithelial cells in nontumor and tumor breast tissue. There was a statistically significant difference in the mean H-score of nuclear GCR among CK5/6 high (mean = 36) and CK5/6 low (mean = 19) samples. Multivariate logistic regression of high CK 5/6 regressed on high GCR while adjusting for race, age at diagnosis and stage, grade, and histological category revealed that high GCR expression remained associated with CK5/6 expression (OR 3.3; 95% CI, 1.6–6.9). CK 5/6 was not associated with race/ethnicity, age at diagnosis, hormone receptor status, stage and grade at diagnosis, or breast cancer subtypes.

## 4. Discussion

Several genetic variants were associated with later disease stage, higher grade, and hormone receptor-negative status even after correction for population stratification, before adjustment for multiple comparisons. Two functional SNPs in GCR (rs6191, rs33388) were associated with a higher grade between White and Black cases, but not Hispanic cases. The minor allele associated with the phenotype differed between the racial/ethnic groups. The minor allele G of rs6191 was associated with an increased prevalence of high grade among White cases, while the minor allele T was associated with a higher grade among Black cases. The minor allele T of rs33388 was associated with an increased prevalence of high grade among White cases, while the minor allele A was associated with a higher grade among Black cases. The rs41423247 variant in GCR was associated with lower grade in the White cases and it has been shown to be associated with hypersensitivity to glucocorticoids [35].

The rs9324924 was associated with a higher grade in the Black and Hispanic cases. However, the minor G allele in Black and the GT and TT genotypes among Hispanics were associated with a higher grade. The rs10482616GA+AA was associated with ER or PR receptor positivity among Black cases. It is hard to interpret the impact of these variants on breast cancer characteristics as none of these SNPs have been previously studied in breast cancer.

We observed an inverse relationship between stage at diagnosis and 3 FKPB5 SNPs (rs3800373, rs9296158, rs9470080) among Black cases. FKBP5 is a co-chaperone, which belongs to the immunophilin family. Immunophilins are a large, functionally diverse group of proteins defined by their ability to bind immunosuppressive ligands. FKBP5 expression is highly inducible by glucocorticoids and functions as a negative transcriptional regulator of GCR [36]. In addition, over-expression of FKBP5 impairs nuclear localization of GCR (Binder, 2009). The rs3800373, rs9296158 and rs9470080 FKPB5 SNPs have been associated with a higher FKBP5 expression and a more potent induction of FKBP5 mRNA by cortisol [37]. Romano et al. have observed a low/negative protein expression of FKBP5 among ten breast cancer samples [38]. If these associations are real and not a result of type 1 error, it is possible that these FKBP5 polymorphisms might be reducing GCR activation by inhibiting nuclear translocation. 

We identified associations with two ADIPOQ SNPs (rs1501299 and rs266729) and stage at diagnosis among Hispanic cases. The ADIPOQ rs1501299_CA+AA_ genotypes were protective against the later stage (OR 0.39, 95% CI 0.17-0.87), while the ADIPOQ rs266729_GC+GG_ genotypes were associated with a later stage (OR 3.01, 95% CI 1.35-6.73). The ADIPOQ (rs1501299 CA+AA) was associated with ER or PR receptor positivity among Black cases. These two SNPs have been previously associated with circulating levels of ADIPOQ and breast cancer. Kaklamani et al. have previously shown that the rs1501299 was associated with increased breast cancer prevalence among African American women [39]. The G allele at rs266729 is associated with lower adiponectin levels and obesity [40]. 

Among the White cases, a higher grade at diagnosis was associated with IL-6 rs1800797_AG+AA_ genotype. IL-6 is an inflammatory cytokine where high serum levels of IL-6 have been shown to correlate with poor outcomes in breast cancer patients [41] and several IL6 SNPs have been associated with breast cancer risk and prognosis [42]. 

The B-cell CLL/lymphoma 2 (BCL2) gene encodes an antiapoptotic protein, a critical regulator of programmed cell death. Higher levels of BCL2 expression in breast tumors have been shown to be an independent prognostic factor for improved survival from breast cancer [43]. The BCL2 rs2279115_AA_ was associated with ER or PR receptor positivity. Bachman et al. found that a higher expression of BCL2 was associated with the A-allele, and survival analysis revealed a significant association of the AA genotype with improved survival [44].

It is possible that those SNPs are not causal; it is also possible that causal SNPs exist but are not located in the measured SNP's vicinity. Given the modest sample sizes within racial and ethnic groups and the large number of SNPs analyzed, genetic variants were not associated with breast cancer characteristics after multiple comparison corrections. This was not unexpected given the limited power to detect associations revealed by post hoc power analyses that generally implied the power to detect associations of 60% or less (not shown). 

Glucocorticoid signaling via GCR regulates many physiological processes, including those involved in mammary development and differentiation. We examined the protein expression of GCR in breast tissue from our breast cancer cases subcohort, BCCC, in a TMA study and compared it to nontumor tissue. We found that GCR was expressed in all normal and fibroadenoma samples and was mainly localized in myoepithelial cells. There was a marked reduction in nuclear GCR expression in breast cancer tissue compared to normal or benign breast tissue lesions that might be due to disruption of the myoepithelial cell layer and basement membrane during tumor invasion [45]. Our findings could reflect either that GCR is involved in a biological pathway leading to breast cancer or is a marker of other causal mechanisms associated with breast cancer development. GCR has been shown to promote both cell survival and cell death, depending on the cell type. High expression of the GCR gene is associated with poor outcomes in ER- patients and better outcomes in ER+ patients [46]. 

Based on our findings, we propose that GCR has a tumor suppressor role in breast cancer. The downregulation of the nuclear GCR observed in our study has also been observed in prostate cancer, another hormone-sensitive tumor [47]. GCR was shown to exert tumor suppressor effects in a skin cancer mouse model [48]. It would be important to compare GCR levels from adjacent histologically normal areas, in situ, and invasive components from the same patient to examine expression changes during breast tumorigenesis.

Several studies from different countries across various ethnic groups have detected both cytoplasmic and nuclear GCR expression using either monoclonal [28,29,30] or polyclonal antibodies against GCR [27]. Our results are in agreement with the pattern of decreased nuclear GCR expression reported in these prior studies. However, we did not observe a decrease in nuclear GCR expression nor increased cytoplasmic GCR with tumor progression [30]. We found that cytoplasmic GCR positively correlated with nuclear GCR expression. Unlike one of these studies [28] we did not find any correlation between GCR expression and age at diagnosis or histological and molecular subtypes of breast cancer. 

We observed a strong correlation between GCR and CK5/6. Cytokeratins are filament-forming proteins that provide mechanical support in epithelial cells [49]. In normal tissue, CK5/6 is mainly expressed in the basal–myoepithelial cell layer of the prostate, breast, and salivary glands. CK5/6 are also seen in benign and malignant tumors of epidermal, squamous mucosal, and myoepithelial origins [50]. The cytokeratins 5/6 are found in the cells of the basal layer of normal breast ducts [51]. Expression of CK 5/6 has been associated with poor breast cancer prognosis and is an independent indicator for shorter relapse-free survival [52]. Furthermore, immunohistochemical expression of basal CK5/6 is associated with aggressive disease and adversely impacts survival in HER2+ breast cancer patients [53]. 

It is difficult to reconcile the correlation between a possible tumor suppressor, GCR, with a marker of an aggressive phenotype of breast cancer, CK 5/6. There might be a functional connection between GCR and the CK 5/6 independent of breast cancer. GCR knockout mice have significant skin development defects with impaired keratinocyte differentiation and aberrant proliferation and apoptosis [54]. 

This study’s strength is that the samples came from a population-based study of breast cancer patients' detailed demographic and clinical data and, therefore, may be generalizable to an urban population. This study’s limitations include its cross-sectional nature, limiting the ability to assess temporal aspects of our associations. There are also limitations in the tissue microarray and immunohistochemical staining technique used in this and other studies. Tissue stained might not represent the tumor due to tumor heterogeneity.

## 5. Conclusions

To the best of our knowledge, this is the first study to examine the relationship between GCR and GCR-related gene polymorphisms, GCR protein expression and breast cancer characteristics. Activation of the glucocorticoid-mediated pathway plays an essential role in several cellular processes, and disruption of GCR activity could play a role in breast cancer progression and aggression. Using samples from an urban, multiracial study of breast cancer, we found several genetic variants of functionally important SNPs associated with later disease stage, higher grade, and hormone receptor-negative status. GCR protein was expressed in all normal and fibroadenoma samples. GCR expression is reduced in breast cancer tissue and correlated with the basal cell marker CK5/6.

## Figures and Tables

**Table 1:** Baseline characteristics of the BCCC sample stratified by self-reported race/ethnicity.

Variables	Total	Whites %	Blacks %	Hispanics %	*p*-Value
**Genetic Ancestry,** Mean (±SD)					
European		90(11)	20(13)	40(20)	<0.0001
West African		10(10)	80(13)	20(20)	<0.0001
Native American		3(5)	4(4)	40(24)	<0.0001
**Age at first birth**, Mean years (±SD)		26(6)	21(5)	23(6)	<0.0001
**Age at last birth**, Mean years (±SD)		31(6)	29(5)	31(6)	<0.0001
**Age at diagnosis**					
<50years	224	32	34	38	0.554
≥50years	433	68	66	62	
**CDC categories of BMI** (kg/m^2^)					
Normal weight (18.5–24.9)	203	49	20	20	<0.0001
Overweight (25.0–29.9)	195	22	29	47	
Obese (≥30.0)	257	29	51	33	
**Education**					
Less than High school	120	4	20	44	<0.0001
High school	138	15	27	21	
Some college	397	81	53	36	
**Annual household Income**					
Less than $30,000	263	17	56	57	<0.0001
$30,000 to $75,000	277	52	38	37	
Greater than $75,000	102	31	6	7	
**Insurance category**					
No outpatient insurance	84	7	14	23	<0.0001
Public	125	4	31	23	
Private	447	89	55	55	
**Any co-morbidities**					
No	286	49	37	48	0.007
Yes	370	51	64	52	
**Nulliparous**					
No	523	63	90	93	<0.0001
Yes	133	37	11	7	
**Menopausal status**					
No	133	17	20	27	0.113
Yes	519	83	80	73	
**Family history breast cancer**					
No	503	75	77	84	0.172
Yes	147	25	23	16	
**Mode of detection**					
Screen-detected	336	60	46	45	0.003
Symptoms and no recent prior screen	156	20	28	23	
Symptoms despite a recent prior screen	164	20	27	32	
**Stage at diagnosis**					
0,1 (early stage)	374	67	55	48	0.0004
2,3,4 (late stage)	269	33	45	53	
**Histologic grade**					
Low/intermediate	409	71	60	70	0.001
High	208	29	40	30	
**ER and/or PR**					
ER and/or PR positive	476	87	72	79	<0.0001
Double negative	126	14	28	21	
**Her2/neu overexpression**					
No	305	90	78	86	0.028
Yes	57	10	22	14	

*p*-values for categorical variables are from χ2 tests and from ANOVA for continuous variables for differences according to self-reported race/ethnicity.

**Table 2:** SNPs with a significant association with a higher grade at diagnosis (*p* < 0.06).

dbSNP ID	Genotype	Low Grade (%)	High Grade (%)	OR High grade (95% CI)	*p*-Value
**White ^a^**					
***GCR* rs6191**					
	T/T	27	30	Ref	0.034
	G/T	53	37	0.65 (0.32–1.3)	
	G/G	20	33	1.66 (0.77–3.58))	
	G/G vs. T/T + G/T	20	33	2.16 (1.13–4.15)	0.021
***GCR* rs33388**					
	A/A	29	31	Ref	0.049
	T/A	51	37	0.68 (0.34–1.35)	
	T/T	20	32	1.67 (0.78–3.55)	
	T/T vs. A/A + T/A	20	32	2.09 (1.09–4.01)	0.028
***GCR* rs41423247**					
	G/G	37	52	Ref	0.052
	G/C	51	34	0.48(0.26–0.89)	
	C/C	12	15	0.91 (0.38–2.17)	
	G/C + C/C vs. G/G	63	49	0.56 (0.32–0.99)	0.046
***IL-6* rs1800797**					
	G/G	42	27	Ref	0.079
	A/G	46	60	2.06 (1.08–3.93)	
	A/A	12	13	1.73 (0.66–4.49)	
	A/G + A/A vs. G/G	58	74	1.99 (1.07–3.73)	0.027
**nH Black ^b^**					
**rs6191**					
	G/G	36	20	Ref	0.002126
	G/T	48	48	1.62 (0.85–3.11)	
	T/T	16	32	3.73 (1.75–7.97)	
***GCR* rs33388**					
	T/T	36	21	Ref	0.005
	T/A	51	51	1.59 (0.84–3)	
	A/A	14	28	3.55 (1.62–7.8)	
	A/A vs. T/T + T/A	14	28	2.63 (1.36–5.11)	0.004
***GCR* rs10052957**					
	G/G	61	42	Ref	0.007
	A/G	35	42	1.56 (0.89–2.74)	
	A/A	4	15	4.61 (1.64–12.97)	
***GCR* rs258813**					
	G/G	54	39	Ref	0.016
	G/A	40	43	1.39 (0.79–2.45)	
	A/A	6	18	3.7 (1.48–9.28)	
	A/A vs. G/G + G/A	6	18	3.16 (1.32–7.59)	0.008
***GCR* rs41423247**					
	G/G	63	48	Ref	0.033
	G/C	32	42	1.76 (1–3.09)	
	C/C	5	10	2.98 (1.06–8.4)	
	G/C + C/C vs. G/G	37	53	1.92 (1.13–3.28)	0.015
***GCR* rs10482616**					
	G/G	62	80	Ref	0.015
	G/A	34	15	0.39 (0.2–0.76)	
	A/A	4	5	0.98 (0.28–3.44)	
	G/A + A/A vs. G/G	38	20	0.46 (0.25–0.84)	0.01
***GCR* rs10482672**					
	C/C	66	81	Ref	0.058
	T/C	28	15	0.43(0.21–0.89)	
	T/T	6	5	0.67 (0.19–2.37)	
	T/C + T/T vs. C/C	34	19	0.47 (0.25–0.91)	0.021
***GCR* rs7701443**					
	A/A	24	39	Ref	0.008
	A/G	56	53	0.56 (0.31–1.01)	
	G/G	20	8	0.26 (0.1–0.65)	
	A/G + G/G vs. A/A	77	61	0.48 (0.27–0.86)	0.013
***GCR* rs9324921**					
	C/C	68	80	Ref	0.067
	C/A	28	17	0.47 (0.24–0.9)	
	A/A	3	3	0.77 (0.17–3.5)	
	C/A + A/A vs. C/C	32	20	0.5 (0.27–0.93)	0.025
***FKBP* rs9296158**					
	G/G	27	30	Ref	0.066
	A/G	47	55	0.96 (0.52–1.78)	
	A/A	26	16	0.44 (0.2–0.98)	
	A/A vs. G/G + A/G	26	16	0.45 (0.23–0.9)	0.02
**Hispanic cases ^c^**					
***GCR* rs9324924**					
	G/G	33	13	Ref	0.075
	G/T	45	67	3.59 (1.09–11.82)	
	T/T	22	20	2.22 (0.54–9.1)	
	G/T + T/T vs. G/G	67	87	3.14 (0.99–10)	0.036

^a^ Adjusted for European genetic ancestry, health insurance, income, education, nulliparity, and age at first and last birth. ^b^ Adjusted for West African genetic ancestry, health insurance, income, education, nulliparity, and age at first and last birth. ^c^ Adjusted for Native American genetic ancestry, health insurance, income, education, nulliparity, and age at first and last birth.

**Table 3:** List of SNPs with significant association with later stage at diagnosis (*p* < 0.06).

dbSNP ID	Genotype	Early Stage (%)	Late Stage (%)	OR Late Stage (95% CI)	*p*-Value
**nH Black ^a^**					
***GCR* rs10482614**					
	G/G	65	62	Ref	0.005
	A/G	34	29	0.8 (0.5–1.4)	
	A/A	1	9	7.96 (1.6–38.9)	
***FKBP5* rs3777747**					
	A/A	36	38	Ref	0.130
	A/G	52	43	0.84 (0.49–1.46)	
	G/G	12	19	1.82 (0.3–3.98)	
	G/G vs. A/A−A/G	12	19	2.01 (0.98–4.11)	0.054
***FKBP55* rs3800373**					
	T/T	30	42	Ref	0.054
	G/T	52	41	0.51(0.29–0.91)	
	G/G	18	17	0.52 (0.24–1.12)	
	G/T−G/G vs. T/T	70	58	0.51(0.3–0.89)	0.016
***FKBP55* rs9296158**					
	G/G	23	35	Ref	0.044
	A/G	56	44	0.47 (0.26–0.86)	
	A/A	21	21	0.54 (0.26–1.13)	
	A/G−A/A vs. G/G	77	65	0.49 (0.28–0.87)	0.014
***FKPB5* rs9470080**					
	C/C	24	38	Ref	0.042
	C/T	57	46	0.47 (0.25–0.87)	
	T/T	19	16	0.47 (0.21–1.08)	
	C/T−T/T vs. C/C	76	62	0.47 (0.26–0.85)	0.012
**Hispanic ^b^**					
***ADIPOQ* rs1501299**					
	C/C	37	57	Ref	0.054
	C/A	50	33	0.35 (0.15–0.84)	
	A/A	13	10	0.54 (0.15–1.94)	
	C/A−A/A vs. C/C	63	43	0.39 (0.17–0.87)	0.019
***ADIPOQ* rs266729**					
	C/C	70	43	Ref	0.012
	G/C	26	43	2.55 (1.09–5.97)	
	G/G	4	13	6.47 (1.2–34.83)	
	G/C−G/G vs. C/C	30	57	3.01(1.35–6.73)	0.006

^a^ Adjusted for West African genetic ancestry, health insurance, income, education, nulliparity, and age at first and last birth. ^b^ Adjusted for Native American genetic ancestry, health insurance, income, education, nulliparity, and age at first and last birth.

**Table 4:** List of SNPs with significant association with hormone receptor positivity (ER or PR) (*p* < 0.06).

dbSNP ID	Genotype	HR Positive (%)	HR Negative (%)	OR HR Positivity (95% CI)	*p*-Value
**nH White cases ^a^**					
***GCR* rs12656106**					
	G/G	40	35	Ref	0.025
	C/G	24	48	2.24(0.76–6.63)	
	C/C	36	17	0.47 (0.17–1.35)	
	C/C vs. G/G−C/G	36	17	0.32(0.12–0.83)	0.023
**nH Black cases ^b^**					
***GCR* rs10482616**					
	G/G	80	64	Ref	0.142
	G/A	17	31	2.05 (0.94–4.49)	
	A/A	3	5	1.93(0.39–9.67)	
	G/A−A/A vs. G/G	20	36	2.03 (0.98–4.21)	0.04828
***ADIPOQ* rs1501299**					
	C/C	56	41	Ref	0.07604
	C/A	38	47	1.82 (0.95–3.47)	
	A/A	7	12	2.78 (0.85–9.1)	
	C/A−A/A vs. C/C	44	59	1.96 (1.06–3.63)	0.03116
***BCL2* rs2279115**					
	C/C	53	57	Ref	0.02856
	C/A	42	27	0.6 (0.31–1.16)	
	A/A	5	16	2.84 (0.78–10.28)	
	A/A vs. C/C−C/A	5	16	3.48 (0.99–12.24)	0.02787

^a^ Adjusted for European genetic ancestry, health insurance, income, education, nulliparity, and age at first and last birth. ^b^ Adjusted for West African genetic ancestry, health insurance, income, education, nulliparity, and age at first and last birth.

**Table 5:** Summary of significant associations between genetic variants and breast cancer characteristics.

Breast Cancer Characteristics						
	Grade		Stage		ER/PR	
Self-Reported race/ethnicity	High Grade	Low Grade	Late Stage	Early Stage	ER or PR Positive	ER and PR Negative
**nH White**	***GCR*** rs33388 rs6191	***GCR*** rs41423247				***GCR*** rs12656106
	***IL-6*** rs1800797	-				
**nH Black**	***GCR*** rs33388 rs6191 rs10052957 rs258813 rs2918418 rs41423247 rs9324924	***GCR*** rs10482616 rs10482672 rs7701443 ***FKBP5*** rs9296158	***GCR*** rs10482614 ***FKBP5*** rs3777747	***FKBP5*** rs3800373 rs9296158 rs9470080	***GCR*** rs10482616, ***ADIPOQ*** rs1501299 *BCL2* rs2279115	
**Hispanic**	***GCR*** rs9324924		***ADIPOQ*** rs266729	***ADIPOQ*** rs1501299		

**Table 6:** GCR expression in breast tissue from the BCCC TMA study.

	N	GCR
		% High GCR ^a^
**Reduction mammoplasty**	26	100
**Fibroadenoma**	5	100
**Breast cancer**	253	44
		*p* < 0.0001 ^b^
**Histological breast cancer subtypes**		
Ductal carcinoma	191	42
Lobular carcinoma	29	48
Mixed & Other	33	19
		*p* = 0.355 ^b^
**Molecular breast cancer subtypes**		
Luminal A	175	42
Luminal B	14	57
Triple Negative	45	47
Her2	21	29
		*p* = 0.353 ^b^
**Hormone receptor status**		
ER+ and/or PR+	177	44
ER− and PR−	61	44
		*p* = 0.21 ^b^
**CK5/6 status**		
Low	163	47
High	93	53
		*p* < 0.000 ^b^

^a^ Percentage positive: a tissue was considered high positive for nuclear GCR when the sample had an H-score >17, ^b^ Chi-square *p*-value.

## Data Availability

Data presented in this study are available on request from the first author (UA).

## Appendix A

**Table A1:** List of antibodies for immunohistochemical staining.

Antigen	Manufacturer	Host	Clone #	Dilution	Retrieval Method
**GCR**	**Leica/Novocastra**	**Mouse**	**4H2**	**1:25**	**HIER**
**IHC subtyping antibodies panel:**					
**ER**	Ventana	Rabbit	SP1	Predilute	CC1 Mild
**PR**	Ventana	Rabbit	1.00E + 02	Predilute	CC1 Mild
**Her-2**	Ventana	Mouse	4B5	Predilute	CC1 Mild
**CK 5/6**	DAKO	Mouse	D5 & 16B4	1:50	HIER
**EGFR**	Ventana	Mouse	3C6	Predilute	CC1 Mild

Abbreviations: HCF: UIC Histology Core Facility; MC: Medical Center at Chicago Clinical Reference Surgical Pathology laboratory; HIER: Heat-induced epitope retrieval; CC1: cell conditioning solution 1.

**Table A2:** Characteristics of studied polymorphism.

**dbSNP_ID**	**Coordinates**	**Type**	**nH Blacks**				**nH Whites**			**Hispanics**		
			**MA**	**MAF**		**HWE**	**MA**	**MAF**	**HWE**	**MA**	**MAF**	**HWE**
	**GCR (*NR3C1*) at 5q31.1**											
rs174048	142650404	upstream of NR3C1	C	0.167		1.00	C	0.164	0.48	C	0.09	1.0
rs17287745	142655015	Downstream gene	G	0.135		0.11	G	0.369	0.10	G	0.26	0.2
rs17287758	142657021	Downstream gene	A	0.080		0.68	A	0.155	0.63	A	0.08	1.0
rs6191	142658156	3′ UTR	T	0.456		0.63	G	0.482	1.00	T	0.38	0.4
rs17209251	142669223	Intron	G	0.055		0.17	G	0.192	0.68	G	0.17	0.1
rs258813	142674690	Intron	A	0.306		0.77	A	0.322	0.47	A	0.18	0.4
rs6188	142680344	Intron	T	0.272		0.16	T	0.290	0.005	T	0.14	1.0
rs10482672	142692533	Intron	T	0.175		0.006	T	0.168	0.22	T	0.10	0.1
rs33388	142697295	Intron	A	0.443		0.62	T	0.476	0.61	A	0.38	0.7
rs2918418	142723373	Intron	G	0.197		0.44	G	0.170	0.18	G	0.10	0.6
rs4912905	142730376	Intron	C	0.101		0.32	C	0.206	0.56	C	0.27	0.2
rs2963155	142756004	Intron	G	0.279		0.65	G	0.260	0.19	G	0.15	1.0
rs9324921	142767740	Intron	A	0.153		0.35	A	0.059	0.19	A	0.05	1.0
rs41423247	142778575	Intron	C	0.251		0.52	C	0.353	1.00	C	0.25	0.1
rs6195	142779317	Exon	G	0.009		1.00	G	0.026	1.00	G	0.00	1.0
rs6189	142780339	Synonymous	0	0.000		1.00	A	0.036	0.27	A	0.01	0.004
rs10482616	142781567	Intron	A	0.182		0.41	A	0.170	1.00	A	0.25	0.8
rs10482614	142782402	Intron	A	0.204		0.71	A	0.169	0.11	A	0.12	1.0
rs10482605	142783521	Intron	C	0.054		0.003	C	0.091	0.0001	C	0.04	0.1
rs72801094	142785905	Intron	C	0.019		1.00	C	0.060	0.60	C	0.05	1.0
rs10052957	142786701	Intron	A	0.273		0.76	A	0.320	0.67	A	0.20	1.0
rs9324924	142792484	Intron	G	0.338		0.68	T	0.421	1.00	T	0.46	0.9
rs7701443	142792650	Intron	G	0.431		0.11	G	0.406	1.00	G	0.48	0.7
rs4244032	142794725	Intron	G	0.135		0.80	G	0.169	1.00	G	0.11	0.6
rs4607376	142796532	Intron	G	0.241		0.03	A	0.480	0.16	G	0.44	0.3
rs13182800	142801480	Intron	T	0.194		0.07	T	0.183	0.40	T	0.12	0.2
rs12054797	142805902	Intron	T	0.097		0.02	T	0.248	0.86	T	0.28	0.5
rs12656106	142808947	Intron	C	0.167		0.64	C	0.407	0.33	C	0.34	0.4
rs12521436	142817607	Upstream gene	A	0.236		0.61	A	0.167	0.25	A	0.220	0.006
rs4912913	142818306	Upstream gene	T	0.5		0.05	T	0.476	0.16	C	0.333	0.01
	***FKBP5* at 6p21.3-21.2**											
rs3800373	35542476	3′ UTR	G	0.409		0.61	G	0.260	1.00	G	0.34	0.8
rs1360780	35607571	Intron	T	0.321		0.00001	T	0.172	0.04	T	0.14	0.02
rs17614642	35621921	Intron	C	0.023		1.00	C	0.077	0.16	C	0.06	0.4
rs34866878	35544942	Synonymous	A	0.174		0.83	A	0.030	1.00	A	0.07	0.5
rs3777747	35579002	Intron	G	0.389		1.00	G	0.488	0.70	G	0.47	1.0
rs3798346	35562640	Intron	G	0.044		0.32	G	0.175	1.00	G	0.11	1.0
rs4713916	3566983	Intron	A	0.134		0.28	A	0.270	0.52	A	0.25	0.1
rs9296158	35567082	Intron	A	0.464		0.81	A	0.304	0.77	A	0.37	0.8
rs9470080	35646435	Intron	T	0.435		0.51	T	0.294	0.19	T	0.37	1.0
rs41270080	35542045	3′ UTR	A	0.170		0.34	A	0.036	1.00	A	0.07	1.0
rs4713899	35569281	Intron	A	0.133		0.59	A	0.165	0.23	A	0.12	0.1
rs6912833	35617585	Intron	A	0.169		1.00	A	0.280	0.64	A	0.27	1.0
rs737054	35575487	Noncoding exon	T	0.078		0.21	T	0.282	0.88	T	0.29	1.0
rs73746499	35578851	Intron	G	0.182		1.00	G	0.032	1.00	G	0.08	0.6
rs755658	35549670	3 prime UTR	A	0.023		1.00	A	0.085	0.69	A	0.14	0.7
rs9366890	35562974	Intron	T	0.175		0.13	T	0.178	0.83	T	0.13	0.1
rs9380524	35589070	Intron	A	0.025		1.00	A	0.094	0.14	A	0.09	1.0
	***SGK-1* 6q23.2**											
rs9493857	134530697	Intron	G	0.272		0.06	A	0.201	0.0000	A	0.41	0.2
	***BCL2* 18q21.33**											
rs2279115	60986837	5' UTR	A	0.260		0.002	C	0.451	0.52	C	0.36	0.1
	***IL-6* 7p21**											
rs1800796	22766246	Noncoding exon	A	0.130		0.09	A	0.379	1.00	A	0.21	1.0
rs1800797	22766221	Noncoding exon	A	0.091		0.03	A	0.054	0.42	A	0.18	0.3
rs1800795	22766645	Intron	C	0.112		0.47	C	0.378	1.00	C	0.19	1.0
rs6949149	22749157	Regulatory region	T	0.123		0.002	T	0.088	0.42	T	0.18	0.1
	***ADIPOQ* 3q27.3**											
rs266729	186559474	Upstream gene	G	0.119		0.78	G	0.276	0.15	G	0.26	0.3
rs1501299	186571123	Intron	A	0.321		0.78	A	0.268	0.52	A	0.33	0.7
	***LEPR 1p31.3***											
rs1137100	66036441	Exon	G	0.182		0.41	G	0.230	0.21	G	0.30	0.8
rs1137101	66058513	Exon	A	0.494		0.72	G	0.434	0.12	G	0.44	0.3
	***SOD2* 6q25.3**											
rs4880	160113872	Missense	C	0.489		0.40	T	0.500	0.38	T	0.38	0.8
	***CAT* 11p13**											
rs1001179	34460231	Upstream gene	A	0.061		1.00	A	0.244	0.287	A	0.066	1.0

Abbreviations: MA: minor allele, MAF: minor allele frequency, HWE: Hardy−Weinberg equilibrium, UTR: untranslated region. DNA position: according to the NCBI genomic reference sequence NT_029289.11.

**Table A3:** SNPs removed from analysis.

Self-Reported Race/Ethnicity	Black	White	Hispanic
Excluded SNPs	rs6189 rs6195 rs1360780 rs755658 rs9380524 rs17614642 rs72801094	rs6189 rs6195 rs737499 rs1800796 rs9493857 rs5871845 rs9380524 rs10482605 rs34866878 rs41270080 rs7280109	rs6189 rs6195 rs174048 rs9324921 rs2918418 rs1001179 rs1360780 rs10482605 rs17287758 rs41270080 rs72801094

**Table A4:** Distribution of demographic and clinical characteristics of BCCC sub-cohort.

	Total	Whites %	Blacks %	Hispanics %	*p*-Value
**Genetic Ancestry, Mean (±SD)**					
European		88 (12)	16 (12)	48 (20)	<0.0001
West African		9 (11)	80 (12)	15 (19)	<0.0001
Native American		3 (3)	4 (4)	37 (20)	<0.0001
**Age at first birth**, Mean years (±SD)		26 (6)	20 (5)	23 (5)	<0.0001
**Age at last birth**, Mean years (±SD)		31 (6)	29 (5)	32 (6)	0.0129
Age at diagnosis					
<50years	66	29	39	32	0.765
≥50years	149	33	40	28	
**CDC categories of BMI (kg/m^2^)**					
Normal weight (18.5—24.9)	47	40	36	23	0.078
Overweight (25.0—29.9)	75	29	32	39	
Obese (≥30.0)	91	28	48	24	
Education					
Less than High school	55	6	35	60	<0.0001
High school	53	23	55	23	
Some college	107	50	35	16	
**Annual household Income**					
Less than $30,000	102	13	49	38	<0.0001
$30,000 to $75,000	69	44	33	23	
Greater than $75,000	39	56	26	18	
Insurance category					
No outpatient insurance	28	11	50	39	<0.0001
Public	47	6	55	38	
Private	134	44	31	25	
Any comorbidities					
No	94	31	34	35	0.007
Yes	115	31	44	25	
**Nulliparous**					
No	34	74	18	9	<0.0001
Yes	175	23	43	34	
**Menopausal status**					
No	34	21	38	41	0.167
Yes	180	33	40	27	
**Family history breast cancer**					
No	201	31	39	30	0.41
Yes	13	46	39	15	
**Mode of detection**					
Screen-detected	92	44	33	24	0.003
Symptoms and no recent prior screen	59	24	51	25	
Symptoms despite a recent prior screen	58	19	38	43	
**Stage at diagnosis**					
0,1 (early stage)	94	42	32	27	0.021
2,3,4 (late stage)	120	24	46	30	
**Histologic grade**					
Low/intermediate	134	35.1	34.3	30.6	0.068
High	75	25.3	50.7	24	
**ER and/or PR**					
ER and/or PR positive	143	34	36	30	0.203
Double negative	47	26	51	23	
**Her2/neu overexpression**					
No	124	33	36	31	0.068
Yes	23	26	61	13	

*p*-values for categorical variables are from χ2 tests and from ANOVA for continuous variables for differences according to self-reported race/ethnicity.
